# Moderate and deep sedation for non-invasive paediatric procedures in tertiary maternity and children’s hospitals in China: a questionnaire survey from China

**DOI:** 10.1186/s12913-019-4885-4

**Published:** 2020-01-08

**Authors:** Bo Li, Ruidong Zhang, Yue Huang, Kan Zhang, Chun Yin Wat, Jie Bai, Mazhong Zhang, Jijian Zheng

**Affiliations:** 1 Department of Anaesthesiology, Shanghai Children’s Medical Center, Affiliated with Shanghai Jiao Tong University School of Medicine, National Children’s Medical Center, 1678 Dong Fang Road, Shanghai, 200127 China; 2 Department of Anaesthesiology and Perioperative Medicine, Hong Kong Children’s Hospital, 1 Shing Cheong Road, Kowloon, Hong Kong; 3 Pediatric Clinical Pharmacology Laboratory, Shanghai Children’s Medical Center, National Children’s Medical Center, 1678 Dongfang Road, Shanghai, China

**Keywords:** Child, China, Conscious sedation, Deep sedation, Questionnaires, Surveys

## Abstract

**Background:**

Moderate and deep sedation are well-established techniques in many developed countries, and several guidelines have been published. However, they have received attention in China only in recent years. The aim of this study is to investigate current paediatric sedation practices in tertiary children’s hospitals and tertiary maternity and children hospitals in China.

**Methods:**

All tertiary children’s hospitals and tertiary maternity and children hospitals registered with the National Health Commission of the People’s Republic of China were invited to participate in an electronic survey, which included information on the sedation caseload, facility availability, staff structure, clinical skill requirements for sedation providers, fasting guidelines, patient-monitoring practices, and choice of sedatives.

**Results:**

Fifty-eight of the 63 hospitals that completed the survey (92.1%) provided moderate and deep sedation. Dedicated sedation rooms and post-sedation recovery rooms were found in 14 (24.1%) and 19 (32.8%) hospitals, respectively. Sedation for non-invasive procedures was primarily performed by anaesthesiologists (69.0%); however, 75.9% of the sedation providers had not received paediatric basic or advanced life-support training. Children were asked to fast from clear liquids for at least 2 h in 44.8% of hospitals and up to 6 h in 5.2% of hospitals; they were asked to fast from solid food/milk for at least 4 h in 27.6% of hospitals and more than 8 h in 1.7% of hospitals. The most commonly used sedative in all groups was chloral hydrate. For rescue, propofol was the most widely used sedative, particularly for children older than 4 years.

**Conclusions:**

Moderate and deep sedation practices vary widely in tertiary children’s hospitals and tertiary maternity and children hospitals in China. Optimised practices should be established to improve the quality of moderate and deep sedation.

## Background

Moderate and deep sedation techniques play an important role in facilitating paediatric diagnostic procedures such as computed tomography, magnetic resonance imaging, and echocardiography. They assist in diagnostic procedures for uncooperative children and reduce procedure-related stress for cooperative children. In the past 30 years, various guidelines have been published in recognition of the potential risks associated with procedural sedation. In 1985, the American Academy of Pediatrics (AAP) published the first guideline to address safety concerns [1]. Subsequently, the American Society of Anesthesiologists (ASA), the American Academy of Pediatric Dentistry (AAPD), and the American College of Emergency Physicians (ACEP) developed sedation guidelines for different clinical situations [2–4]. These have been updated regularly to enhance the safety and quality of moderate and deep sedation practices in the United States and other developed countries [5–10].

Sedation practices in China are not uniform, possibly due to variability in the workforce, facilities, knowledge, and practice guidelines. There is currently no standardised national practice guideline for procedural sedation in China, and training for sedation providers also varies. Therefore, we aim to investigate current sedation practices in tertiary children’s hospitals and tertiary maternity and children hospitals in China, in the hope of contributing to the future establishment of paediatric sedation guidelines in China for moderate and deep sedation.

## Methods

An electronic questionnaire was developed to explore the current moderate and deep sedation practices for non-invasive diagnostic procedures in Chinese tertiary children’s hospitals and tertiary maternity and children hospitals. Verbal consent was approved by the Institutional Review Board of Shanghai Children’s Medical Centre since our study neither involved the privacy issues of patients nor involved the alterations of paediatric sedation practices (SCMCIRB-W2019018). We identified tertiary children’s hospitals and tertiary maternity and children hospitals from a list of facilities registered with the National Health Commission of People’s Republic of China. We then contacted the departments of anaesthesiology to determine their willingness to participate in this survey. If sedation was not provided by anaesthesiologists, the person in charge of the sedation service was asked to participate. After oral informed consent was obtained, we created a WeChat Sedation Survey Group using a smartphone and generated the survey questionnaire through the built-in WeChat Mini Program. To avoid multiple replies from the same hospital, only one physician familiar with the sedation service from each hospital was invited to join the WeChat Sedation Survey Group. The questionnaires were released in June 2018, and completed surveys were retrieved by July of the same year. The definitions of light sedation, moderate sedation, and deep sedation were indicated in the questionnaire, and it was made clear that the survey questions did not pertain to light sedation. Light sedation was defined as a drug-induced state during which patients respond normally to verbal commands; although cognitive function and coordination may be impaired, ventilatory and cardiovascular functions are unaffected. The questions in the survey were related to the frequency of moderate- and deep-sedation use, facilities, staff structure, prerequisite skills, fasting practices, monitoring practices, and choice of sedatives.

Statistical analysis was performed using SPSS Version 19.0 (SPSS Inc., College Station, TX, USA). For descriptive analyses, categorical variables were reported as counts and percentages. The variability of data was analysed using a chi-squared test or Fisher’s exact test; *p*-values < 0.05 were considered statistically significant.

## Results

We asked 81 tertiary children’s hospitals and tertiary maternity and children hospitals to join the study; 17 did not respond, and 64 agreed to participate. Questionnaires were sent to representatives from each of the 64 hospitals, and valid questionnaires were retrieved from 63 (98.4%) of them. At least one hospital in each province and municipality in China was included, except for the Tibet Autonomous Region and the Ningxia Hui Autonomous Region. According to the National Health Commission of People’s Republic of China, there are no tertiary children’s hospitals or tertiary maternity and children hospitals registered in these two regions.

### Frequency of moderate- and deep-sedation use

Of the 63 hospitals, 58 (92.1%) provided moderate and deep sedation for non-invasive paediatric diagnostic procedures. A majority (*N* = 36, 62.1%) of the hospitals performed moderate-to-deep sedation in fewer than 1000 cases per year. Twelve (20.7%) hospitals performed it in 1000–5000 cases per year, and 10 (17.2%) hospitals performed it in more than 10,000 cases per year. The top 10 centres in terms of number of patients served were located in Shanghai, Chongqing, Guangzhou, Zhengzhou, Xuzhou, Hangzhou, Chengdu, Kunming, Qingdao, and Xining.

### Facilities

**Fig. 1:**
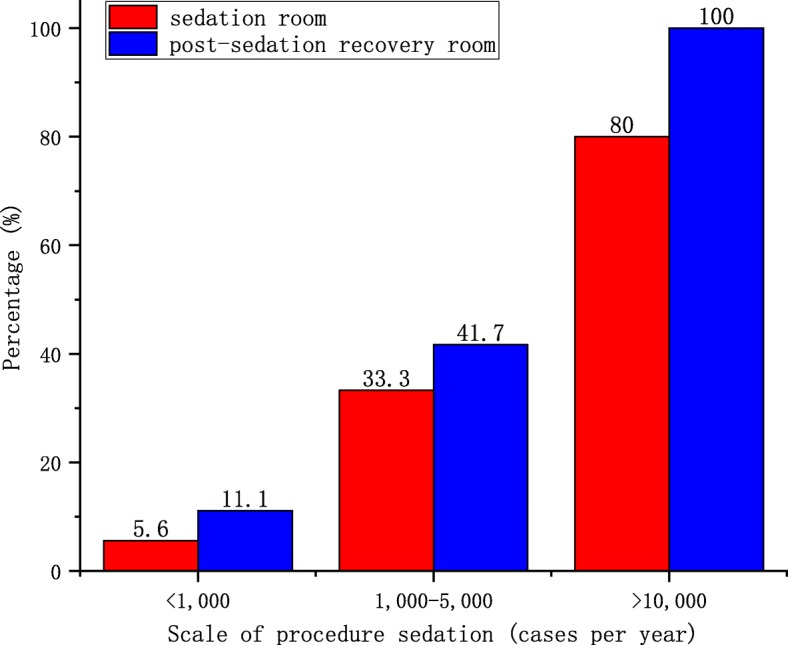
Scale of the facilities providing moderate and deep sedation in China. Red columns: the percentage of sedation rooms. Blue columns: the percentage of post-sedation recovery rooms

Dedicated sedation rooms and post-sedation recovery rooms were reported in only 14 (24.1%) and 19 (32.8%) hospitals, respectively. Of the 10 hospitals with more than 10,000 sedation cases per year, eight (80%) reported dedicated sedation rooms and all (100%) reported post-sedation recovery rooms. The availability of these rooms was less common in hospitals that managed less than 1000 sedation cases per year (*p* < 0.001; Fig. 1).

### Staff structure and prerequisite skills

**Fig. 2:**
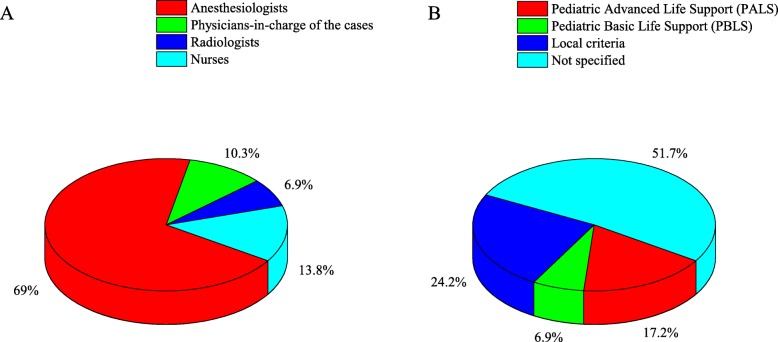
Sedation providers and prerequisite skills for moderate and deep sedation in China. **a**: sedation providers. **b**: the prerequisite skills for sedation providers

Anaesthesiologists performed most sedations (69.0%). Other sedation providers included physicians-in-charge (the first physician to see and care for the patient, 13.8%), radiologists (6.9%), and nurses (10.3%) (Fig. 2). Fifteen (25.9%) hospitals reported that they employed full-time sedation providers, which, in China, are medical personnel whose only duty is to provide sedation service during that session. Of these 15 hospitals, 13 used anaesthesiologists and two used nurses as full-time sedation providers. Ten of the 14 hospitals with sedation rooms had full-time sedation providers; however, only five of the 44 hospitals without sedation rooms had full-time sedation providers (*p* < 0.001).

The ratio of physicians to nurses is shown in Table 1. A ratio lower than 1:1 was reported in 19 (52.8%) hospitals with less than 1000 sedation cases per year and in two (20.0%) hospitals with more than 10,000 sedation cases per year. A ratio equal to or more than 1:4 was reported in two (5.6%) hospitals with less than 1000 sedation cases per year and in four (40%) hospitals with more than 10,000 sedation cases per year. The ratio was not specified in 12 (33.3%) hospitals with less than 1000 sedation cases per year, but all hospitals with more than 10,000 sedation cases per year had explicit requirements regarding the ratio of physicians to nurses.

**Table 1:** Ratio of physicians to nurses for moderate and deep sedation in tertiary children’s hospitals and tertiary maternity and children hospitals in China

Moderate and deep sedation (cases per year)	Hospitals *(n)*	Ratio of physicians to nurses						
		< 1:1	1:1	1:2	1:3	1:4	> 1:4	Not specified
< 1000	36	19	2	0	1	0	2	12
1000-5000	12	1	2	2	0	2	0	5
5000-10,000	0	/	/	/	/	/	/	/
> 10,000	10	2	0	3	1	2	2	0

The prerequisite skills for sedation providers are shown in Fig. 2. Ten (17.2%) hospitals indicated that Paediatric Advanced Life Support (PALS) training is required for staff involved in sedation service, and four (6.9%) hospitals replied that Paediatric Basic Life Support (PBLS) is required. More than half of the hospitals (51.7%) did not specify any training requirements.

### Fasting practices

Solid food or milk was stopped for at least 4, 6, 8, and more than 8 h before sedation in 27.6, 37.9, 25.9, and 1.7% of hospitals, respectively. Clear liquids were stopped for at least 2, 4, and 6 h before sedation in 44.8, 43.1, and 5.2% of hospitals, respectively. Four hospitals (6.9%) did not report pre-sedation fasting requirements (Table 2).

**Table 2:** Pre-sedation fasting requirements in China

Pre-sedation fasting time	Solid food/milk	Clear liquids
2 h	/	44.8%
4 h	27.6%	43.1%
6 h	37.9%	5.2%
8 h	25.9%	/
> 8 h	1.7%	/
Not specified	6.9%	6.9%

### Monitoring practices

Pulse oximetry was used in 65.5 and 77.6% of hospitals during magnetic resonance imaging and non-magnetic procedures, respectively; capnography was used during sedation procedures in approximately 15% of hospitals (Table 3). Most hospitals monitored pulse oximetry in either a continuous or intermittent manner (Table 4).

**Table 3:** Monitoring devices used during moderate and deep sedation in China

Monitoring devices	Percentage of monitoring events used during sedation	
	Magnetic procedures	Nonmagnetic procedures
Pulse oximetry	65.5%	77.6%
Electrocardiography	41.4%	44.8%
Noninvasive blood pressure	27.6%	34.5%
End-tidal carbon dioxide	13.8%	15.5%
Others	12.1%	5.2%

*Notes*: The proportions in Table 3 refer to the percentage of hospitals who use the relevant monitoring event during sedation

**Table 4:** Modes of pulse oximetry monitoring used during moderate and deep sedation in China

Monitoring modes	Percentage of monitoring modes used during sedation	
	Magnetic procedures	Nonmagnetic procedures
Continuous	49.0%	53.7%
Intermittent		
every < 5 min	8.1%	9.2%
every 5–10 min	10.2%	5.6%
every 10–15 min	/	3.7%
every > 15 min	/	1.9%
Not specified	32.7%	25.9%

### Choice of sedatives

**Fig. 3:**
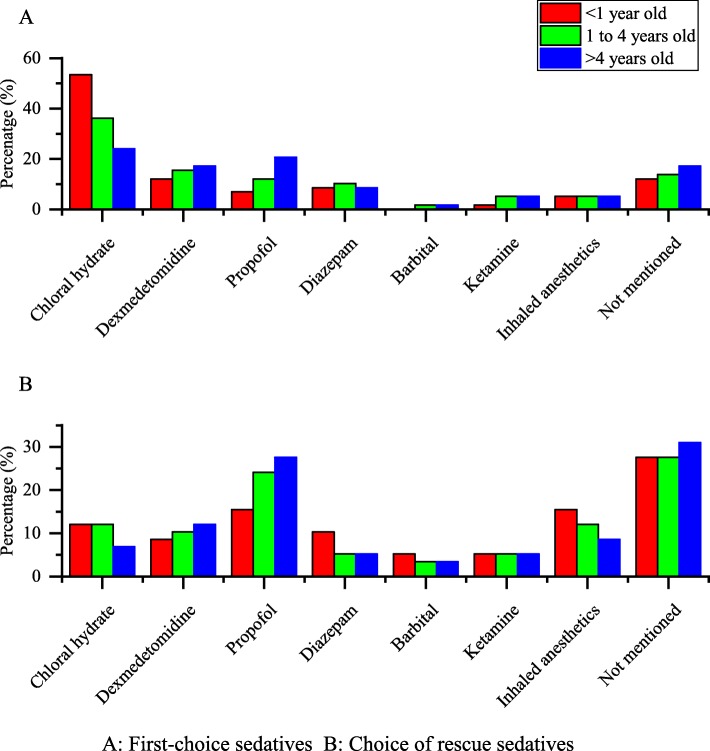
Choice of sedatives for children of different ages in China. **a**: the usage rate of various sedatives as first-choice sedatives in children of different ages. **b**: the usage rate of various sedatives as rescue sedatives in children of different ages. Red columns: children under 1 year old. Green columns: children 1 to 4 years old. Blue columns: children over 4 years old

Chloral hydrate was commonly used as the first-line sedative for children. For infants (younger than 1 year), the three most commonly used sedation agents were chloral hydrate (53.4%), dexmedetomidine (12.1%), and diazepam (8.6%). For older children, chloral hydrate use decreased and dexmedetomidine and propofol use increased. In children older than 4 years of age, the top three sedation agents were chloral hydrate (24.1%), propofol (20.7%), and dexmedetomidine (17.2%) (Fig. 3).

Rescue sedatives were considered by sedation providers if the patient remained awake 30 min after the first-choice agent was administered. The term ‘remained awake’ refers to a Modified Observer’s Assessment of Alertness and Sedation Scale score of ≥4 (Table 5). If the first-choice sedative failed, propofol (15.5%) and inhaled anaesthetics (15.5%) were most commonly used in infants, and an additional dose of chloral hydrate was also considered in 7 hospitals (12.1%). For older children, propofol and dexmedetomidine (instead of inhaled anaesthetics) were popular choices (Fig. 3).

**Table 5:** Modified Observer’s Assessment of Alertness and Sedation Scale (MOAA/S)

Score	Items
0	Dose not respond to a noxious stimulus
1	Dose not respond to mild prodding or shaking
2	Respond only after mild prodding or shaking
3	Respond only after name is called loudly and repeatedly
4	Lethargic respond to name spoken in normal tone
5	Appears asleep but responds readily to name spoken in normal tone
6	Appears alert and awake and responds readily to name spoken in normal tone

## Discussion

Moderate sedation and deep sedation are well-established techniques in many developed countries, and several guidelines have been published. Despite this, these techniques have only recently become prominent in China. Our survey shows that most tertiary children’s hospitals and tertiary maternity and children hospitals in China provide moderate and deep sedation for non-invasive procedures. The hospitals that provide sedation on a larger scale tend to have better facilities and staff composition. Pulse oximetry is monitored in more than 65.5% of hospitals during sedation procedures, but only a few hospitals (about 15%) perform capnography monitoring. Chloral hydrate is the most commonly chosen sedative agent; however, its use tends to decline and the use of dexmedetomidine and propofol increases gradually with patient age. Propofol (15.5%) and inhaled anaesthetics (15.5%) are the most commonly used rescue sedatives. Our study shows that there are no mandatory training requirements for the staff providing sedation in many hospitals.

Most hospitals in China do not have dedicated sedation rooms or post-sedation recovery rooms. Although it is not specifically mentioned in the guidelines, sedation rooms and post-sedation recovery rooms are necessary so that sedation providers can properly monitor and manage paediatric patients. It is alarming that only 24.1 and 32.8% of the hospitals that participated in our survey had sedation rooms and post-sedation recovery rooms, respectively. Considering the potential risks of moderate and deep sedation (e.g. vomiting, aspiration, hypotension, and apnoea), we believe that these standard facilities should be available wherever sedation procedures are provided to ensure the safety of paediatric patients.

According to the National Institute for Health and Care Excellence (NICE) guidelines, staff involved in moderate and deep sedation, including physicians and nurses, should have knowledge of and competency in sedation drug pharmacology, assessment of children, monitoring, recovery care, and management of complications [11]. Moderate sedation and deep sedation require teamwork; although there is no specific requirement for the number of team members, all guidelines emphasize that sedation providers must be competent in life-support skills. The NICE guidelines suggest that all providers of moderate and deep sedation should be trained in basic life-support and at least one team member present for the duration of deep sedation should be competent in advanced life-support [11]. The AAP and AAPD also suggest that at least one team member trained in advanced paediatric life support and skilled in airway management and cardiopulmonary resuscitation be present during deep sedation [6, 7]. It should be noted that although life-support skills are emphasized in all sedation guidelines, no guideline has addressed the question of who should be responsible for sedation. There is no doubt that life-support skills are the specialties of anaesthesiologists; they are also the first choice for sedation management. However, the number of anaesthesiologists is limited and they can hardly fulfil all the needs. Our study reveals that other sedation providers such as nurses and radiologists are also involved. Although accumulating evidences indicate that procedural sedations can be safely and effectively performed in ASA I-II paediatric patients by non-anaesthesiologists, whether this can also be extended to ASA III or higher paediatric patients remains unclear [12–15]. Propofol, a widely used sedative and anaesthetic, has been reported to be safely used for paediatric procedural sedation by non-anaesthesiologists, but whether it can be safely used in paediatric patients with ASA III or higher requires further in-depth investigation [16, 17]. In addition, modified sedative regimens for paediatric patients, such as a combination of ketamine and propofol, also show a reliable safety profile, indicating that it may be an alternative for non-anaesthesiological sedation providers [18, 19]. Anyway, the constant and immediate availability of anaesthesiological support may continue to be necessary for non-anaesthesiological sedation providers [17].

Sedation providers must be competent in life-support skills. If not, someone with life-support skills must be present for the duration of sedation. In view of this point, we think that instead of asking “Who should manage sedation?” we should ask, “What training should sedation providers receive?” According to the guidelines mentioned above, training should include sedation drug pharmacology, assessment of children, monitoring procedures, recovery care, and management of complications. Life-support courses should also be included. PBLS or PALS certification is certainly advantageous; however, it does not mean a provider has been trained to manage sedation. At present, there is no standard training course for sedation providers in China. Specific credentialing and training strategies should be considered in the development of a national guideline. Effective sedation education and training, especially for non-anaesthesiologists, is essential to improve patient safety during moderate and deep sedation. High-fidelity simulation might be an ideal solution; it has been shown to improve skills, knowledge, self-confidence, awareness of emergency, crisis resource management, and teamwork. However, as far as we know, minimal content has been developed for sedation training. Moreover, high-fidelity simulation is costly and requires expertise for the operation of the equipment, which has restricted its popularization.

The NICE guidelines suggest that fasting is not needed for moderate sedation, during which the child maintains verbal contact with the sedation provider. For children who cannot maintain verbal contact (i.e. moderate-to-deep sedation) the 2–4-6 fasting rule should apply [11]. However, it is difficult to accurately control the depth of sedation in clinical practice due to differences in pharmacodynamics and pharmacokinetics. The necessity of pre-sedation fasting has been debated. Many published studies have reported no association between fasting duration and adverse events such as vomiting and pulmonary aspiration [20–22]. Furthermore, the risk of hypovolemia and hypoglycaemia caused by prolonged fasting cannot be ignored in critically ill children; in these cases, a shorter fasting time should be considered. In our institution, milk is allowed 2 h before procedural sedation for children with congenital heart disease. Adverse events such as vomiting and aspiration are extremely rare. It is necessary to have more experienced staff and rescue equipment available to ensure the safety of these children, but we believe that the risks are controllable.

Insufficient monitoring is another problem of moderate and deep sedation in China. Pulse oximetry, electrocardiography, non-invasive blood pressure, and end-tidal carbon dioxide monitoring during moderate and deep sedation is performed in 65.5, 41.4, 27.6, and 13.8% of hospitals during magnetic procedures, respectively, and in 77.6, 44.8, 34.5, and 15.5% of hospitals during nonmagnetic procedures, respectively. This reveals that these monitoring procedures are adopted less frequently than previously reported [23]. End-tidal carbon dioxide monitoring is believed to be more sensitive than pulse oximetry for respiratory events, which can lead to hypoxemia during procedures [24, 25]; the latest ASA guidelines also state that continuous monitoring of ventilatory function with capnography should supplement standard monitoring [9].

The choice of sedatives is another interesting topic. Chloral hydrate was the most commonly used first-choice sedative in our study because of its relatively benign clinical profile and low cost. Other sedatives intended for general anaesthesia (propofol and ketamine) and even inhaled anaesthetics are the first choice in some hospitals. However, 10 years ago, chloral hydrate may have been one of the only options in China (another option may have been barbiturates) for paediatric moderate and deep sedation. There are two reasons for this. First, anaesthesiologists have only recently gradually taken over paediatric moderate and deep sedation practice in China. Sedatives intended for general anaesthesia and inhaled anaesthetics are not considered alternatives if anaesthesiologists are not present. Second, at that time, dexmedetomidine was not yet approved for paediatric clinical use in China, whereas it is now widely used as an ideal sedative. It can be easily administrated intranasally, and children tolerate it well. Miller et al. reported that 2 and 3 μg·kg^− 1^ of intranasal dexmedetomidine were as effective for transthoracic echocardiography sedation as oral chloral hydrate, with similar sedation onset and recovery times in infants and toddlers [26]. Dexmedetomidine can also be used as a rescue sedative. After failed chloral hydrate sedation, the rescue success rate of dexmedetomidine increases in a dose-dependent manner [27], and the effective dose 50 of dexmedetomidine for rescue increases with age in those younger than 3 years [28]. Compared with chloral hydrate, intranasal dexmedetomidine has a higher rescue success rate than a second dose of chloral hydrate after failed chloral hydrate sedation [29]. It should be noted that all references cited in this paragraph are the results of clinical trials for Chinese children. We believe that dexmedetomidine will be more popular as a safe, effective, and well-tolerated sedative in China in the future.

Ketamine is another widely used sedation agent; it can be used safely and effectively for paediatric procedural sedation [30], especially for invasive procedures, because of its analgesic properties [31–33]. Recent studies have reported that ketamine can be used in combination with other sedatives for better sedation quality and fewer adverse events [34–36]. Ketamine seems to be an ideal sedative in most cases; however, only a few providers reported using ketamine as a sedative in our study, probably because it is strictly regulated and difficult to acquire in China.

### Limitations

Although all tertiary children’s hospitals and tertiary maternity and children hospitals registered with the National Health Commission of the People’s Republic of China were asked to participate, 17 hospitals could not be contacted. Some limitations also exist in the study design; only one physician from each hospital was invited to join the survey. Prescribing habits may vary among physicians, other providers (e.g. emergency department doctors) may be involved, and even fasting and monitoring practices may not be strictly enforced after hours. Therefore, responses from one physician may not accurately represent all providers within the institution. Additionally, procedural sedation practices outside of the hospital setting were not captured in our study. All the limitations mentioned above could results in bias. However, the hospitals included in our study did represent the tertiary centres providing paediatric service in different provinces across China, and we believe that the study still sheds light on the state of sedation practice in China.

## Conclusions

Moderate and deep sedation practices, including facilities, staff structure, prerequisite skills, fasting practices, monitoring practices, and choice of sedatives, vary widely among the tertiary children’s hospitals and tertiary maternity and children hospitals in China. Sedation providers in China should work together to established and promulgate evidence-based sedation standards to improve the quality of sedation service in the country.

## Supplementary information

**Additional file 1: Questionnaire**. Description of data: the questionnaire used in our study.

## Abbreviations

AAP: American Academy of Pediatrics
AAPD: American Academy of Pediatric Dentistry
ACEP: American College of Emergency Physicians
ASA: American Society of Anesthesiologists
NICE: National Institute for Health and Care Excellence
PALS: Paediatric Advanced Life Support
PBLS: Paediatric Basic Life Support
