# Supplementary material for: Moderate and deep sedation for non-invasive paediatric procedures in tertiary maternity and children’s hospitals in China: a questionnaire survey from China
Source: BMC Health Serv Res. 2020 Jan 8;20:28. doi: 10.1186/s12913-019-4885-4 (PMC6951005; doi:10.1186/s12913-019-4885-4)
Supplement: Supplementary file 1 — Additional file 1: Questionnaire. Description of data: the questionnaire used in our study. [file 12913_2019_4885_MOESM1_ESM.docx]

Supplementary

**Questionnaire**

1. Name of your institution：
2. How many cases of moderate and deep sedation for non-invasive paediatric procedures are performed in your institution per year?

<1,000

1,000-5,000

5,000-10,000

>10,000

1. Is there a dedicated sedation room in your institution? Yes No
2. Is there a dedicated post-sedation recovery room in your institution? Yes No
3. In your institution, moderate and deep sedation for non-invasive pediatric procedures is performed by:

Anesthesiologists

Physicians-in-charge of the cases

Radiologists

Nurses

Others (please specify):

1. Full-time sedation provider is deployed in your institution: Yes No

(*Notes: a full-time sedation provider is a medical personnel whose only duty is to provide sedation service.*)

1. In your institution, what is the physicians to nurses ratio in sedation service?

<1:1

1:1

1:2

1:3

1:4

>1:4

Not specified

1. In your institution, the prerequisite skills for the sedation staff is/are: (multiple answers allowed)

Pediatric Advanced Life Support (PALS)

Pediatric Basic Life Support (PBLS)

Local criteria

Not specified

Others (please specify):

9. In your institution, solid food/milk (except for breast milk) is stopped for at least __ before sedation:

2 hours

4 hours

6 hours

8 hours

>8 hours

Not specified

10. In your institution, clear liquid is stopped for at least __ before sedation:

2 hours

4 hours

6 hours

8 hours

>8 hours

Not specified

1. In your institution, monitoring device(s) used during moderate and deep sedation for magnetic resonance imaging procedures is/are: (multiple answers allowed)

Pulse oximetry

Electrocardiography

Noninvasive blood pressure

End-tidal carbon dioxide

Others (please specify):

1. In your institution, monitoring device(s) used during moderate and deep sedation for nonmagnetic procedures is/are: (multiple answers allowed)

Pulse oximetry

Electrocardiography

Noninvasive blood pressure

End-tidal carbon dioxide

Others (please specify):

1. In your institution, how was pulse oximetry monitoring being used during moderate and deep sedation for magnetic resonance imaging (MRI)?

Continuous

Intermittent, every <5 min

Intermittent, every 5-10 min

Intermittent, every 10-15 min

Intermittent, every >15 min

Not specified

1. In your institution, how was pulse oximetry monitoring being used during moderate and deep sedation for nonmagnetic procedures?

Continuous

Intermittent, every <5 min

Intermittent, every 5-10 min

Intermittent, every 10-15 min

Intermittent, every >15 min

Not specified

1. In your institution, the first-choice sedative for children under 1 year old is:

Chloral hydrate

Dexmedetomidine

Propofol

Diazepam

Barbital

Ketamine

Inhaled anesthetics

Not specified

Others (please specify):

1. In your institution, the first-choice sedative for children 1 to 4 years old is:

Chloral hydrate

Dexmedetomidine

Propofol

Diazepam

Barbital

Ketamine

Inhaled anesthetics

Not specified

Others (please specify):

1. In your institution, the first-choice sedative for children over 4 years old is:

Chloral hydrate

Dexmedetomidine

Propofol

Diazepam

Barbital

Ketamine

Inhaled anesthetics

Not specified

Others (please specify):

1. In your institution, if sedation effect is not satisfactory after first-choice sedative, the rescue sedative for children under 1 year old is:

Chloral hydrate

Dexmedetomidine

Propofol

Diazepam

Barbital

Ketamine

Inhaled anesthetics

Not specified

Others (please specify):

1. In your institution, if sedation effect is not satisfactory after first-choice sedative, the rescue sedative for children 1 to 4 years old is:

Chloral hydrate

Dexmedetomidine

Propofol

Diazepam

Barbital

Ketamine

Inhaled anesthetics

Not specified

Others (please specify):

1. In your institution, if sedation effect is not satisfactory after first-choice sedative, the rescue sedative for children over 4 years old is:

Chloral hydrate

Dexmedetomidine

Propofol

Diazepam

Barbital

Ketamine

Inhaled anesthetics

Not specified

Others (please specify):
